# Vitamin D-Binding Protein Polymorphisms, 25-Hydroxyvitamin D, Sunshine and Multiple Sclerosis

**DOI:** 10.3390/nu10020184

**Published:** 2018-02-07

**Authors:** Annette Langer-Gould, Robyn M. Lucas, Anny H. Xiang, Jun Wu, Lie H. Chen, Edlin Gonzales, Samantha Haraszti, Jessica B. Smith, Hong Quach, Lisa F. Barcellos

**Affiliations:** 1 Los Angeles Medical Center, Neurology Department, Southern California Permanente Medical Group, 1505 N Edgemont Street, Los Angeles, CA 90027, USA; 2 College of Medicine, Biology & Environment, Australian National University, Canberra, ACT 2000, Australia; robyn.lucas@anu.edu.au; 3 Department of Research & Evaluation, Kaiser Permanente Southern California, 100 S. Los Robles Avenue, Pasadena, CA 91101, USA; anny.h.xiang@kp.org (A.H.X.); jun.x.wu@kp.org (J.W.); lie.h.chen@kp.org (L.H.C.); edlin.g.gonzales@kp.org (E.G.); samanthaha@pcom.edu (S.H.); jessica.b.smith@kp.org (J.B.S.); 4 Philadelphia College of Osteopathic Medicine, 4000 Presidential Blvd., Apt. 819, Philadelphia, PA 19131, USA; 5 QB3 Genetic Epidemiology and Genomics Lab, School of Public Health, University of California Berkeley, 209 Hildebrand Hall, Berkeley, CA 94720, USA; hquach@berkeley.ed (H.Q.); lbarcellos@berkeley.edu (L.F.B.)

**Keywords:** multiple sclerosis, vitamin D, polymorphisms, blacks, Hispanics

## Abstract

Blacks have different dominant polymorphisms in the vitamin D-binding protein (DBP) gene that result in higher bioavailable vitamin D than whites. This study tested whether the lack of association between 25-hydroxyvitamin D (25OHD) and multiple sclerosis (MS) risk in blacks and Hispanics is due to differences in these common polymorphisms (rs7041, rs4588). We recruited incident MS cases and controls (blacks 116 cases/131 controls; Hispanics 183/197; whites 247/267) from Kaiser Permanente Southern California. AA is the dominant rs7041 genotype in blacks (70.0%) whereas C is the dominant allele in whites (79.0% AC/CC) and Hispanics (77.1%). Higher 25OHD levels were associated with a lower risk of MS in whites who carried at least one copy of the C allele but not AA carriers. No association was found in Hispanics or blacks regardless of genotype. Higher ultraviolet radiation exposure was associated with a lower risk of MS in blacks (OR = 0.06), Hispanics and whites who carried at least one copy of the C allele but not in others. Racial/ethnic variations in bioavailable vitamin D do not explain the lack of association between 25OHD and MS in blacks and Hispanics. These findings further challenge the biological plausibility of vitamin D deficiency as causal for MS.

## 1. Introduction

Exposure to ultraviolet radiation (UVR) from sun exposure stimulates intradermal synthesis of vitamin D and is the principal natural source of this vitamin. Higher lifetime UVR has been associated with a decreased risk of multiple sclerosis (MS) in whites [1,2,3,4], and recently we reported similar findings in blacks and Hispanics. However, higher serum 25-hydroxyvitamin D (25OHD) levels [1,4,5,6] are associated with a reduced risk of MS only in whites. We and others have been unable to detect an association between serum 25OHD levels and MS risk in blacks [5,7] or Hispanics [7]. Whether this is because serum 25OHD is not a good indicator of vitamin D status in non-whites or due to the many vitamin D-independent immunological effects of UVR is unknown.

Blacks and Hispanics have lower 25OHD levels than whites, yet there is substantial evidence that low 25OHD levels in blacks do not lead to any excess ill health consequences [8]. Recent studies of the general population have argued that total serum 25OHD levels are a poor indicator of vitamin D status in blacks and that bioavailable or ‘free’ 25OHD and/or its metabolites may be superior indicators [8,9]. Blacks have different dominant common polymorphisms in the vitamin D-binding protein gene than whites that directly affect binding avidity and bioavailability of vitamin D metabolites. Vitamin D-binding protein (DBP), among other functions, is the main transporter of 25OHD to target tissues [10]. Thus, it is biologically plausible that differences in these DBP polymorphisms could explain why we were unable to detect an association between 25OHD and MS risk in blacks and Hispanics.

The primary purpose of the MS Sunshine study was to test the vitamin D-MS hypothesis in blacks and Hispanics including variations in the gene that encodes DBP. The results presented herein address whether high serum 25OHD, independent of UVR, reduces the risk of MS in those individuals that carry the common DBP polymorphisms that are dominant in whites, regardless of race/ethnicity.

## 2. Materials and Methods

### 2.1. Study Population

Participants in the MS Sunshine Study were recruited from the Kaiser Permanente Southern California (KPSC) membership between December 2011 and December 2014 via mailings and telephone contact. KPSC is a large prepaid health maintenance organization with over 4 million members representative of the general population in Southern California [11]. KPSC uses an integrated electronic health record (EHR) system which includes all inpatient and outpatient encounters, diagnostic tests, diagnoses and medications, as well as some demographic and behavioral characteristics. Data were collected from the EHR and, after informed consent, structured in-person interview and a blood draw for genotyping and 25OHD measurement (if not already available in the EHR). All subjects gave their informed consent for inclusion before they participated in the study. The study was conducted in accordance with the Declaration of Helsinki, and the protocol was approved by the KPSC institutional review board (IRB 5962).

#### 2.1.1. Case Identification

Incident cases with MS or clinically isolated syndrome (CIS) were identified using similar methods as previously described [12,13]. Briefly, we searched EHRs monthly for first mention of ICD-9 diagnostic codes for MS or CIS. Diagnoses were confirmed by an MS specialist (ALG) according to diagnostic criteria/consensus definitions for MS [14] or CIS [15,16]. Eligibility required diagnosis of MS or CIS within the past 1.5 years or symptom onset within the past 3 years and age ≥18 years (see Appendix A).

#### 2.1.2. Control Selection

Once a case interview was completed, at least 1 control participant from the KPSC population, matched to the case on race/ethnicity, date of birth (within 2 years), sex and home KPSC facility (a surrogate measure for socioeconomic status) was identified from the EHR and recruited. The controls were assigned the same index date as their matched case (symptom onset date).

### 2.2. Data Collection

Self-identified race/ethnicity was obtained from the interview. White, non-Hispanics were classified as white; any black race regardless of ethnicity was classified as black; and those who identified themselves as white, Hispanics were classified as Hispanics. Comparison with genetic ancestry markers validated the accuracy of self-identified race/ethnicity.

Covariates obtained from the interview included sun exposure during weekends and holidays (leisure time) age 6 years to date of interview, places of residence since birth, smoking (never/ever) and vitamin D supplement use defined as ≥600 IU daily at or within 3 months prior to 25OHD measurement. Age was defined as age at index date. Body mass index (BMI) closest to the date of 25OHD measurement was obtained from EHR.

Total serum 25OHD was measured using liquid chromatography, tandem mass spectrometry. The sensitivity of the assay is <2.5nmol/L. The intra-and inter-assay coefficients of variation are less than 5·2% at 25, 62·5 and 192·5 nmol/L.

### 2.3. Genotyping

DNA samples were genotyped for two common single-nucleotide polymorphism (SNPs) in the coding region of the vitamin D-binding protein gene (rs7041 and rs4588) located on chromosome 4. These SNPs were selected because they are established markers of DBP phenotype and vary by race [8]. HLA-DRB1*15:01 status was determined using a tag SNP (rs3135388). Genetic ancestry was determined with the software STRUCTURE Version 2.3.1, University of Oxford, Oxford, United Kingdom [17] using a genome-wide set of 67,547 linkage disequilibrium pruned loci selected by PLINK 1.07, Center for Human Genetic Research, the Broad Institute of Harvard & MIT, Boston, USA [18] (see Appendix A).

### 2.4. Statistical Analysis

#### 2.4.1. Sun Exposure

We used the most rigorous method available for assessing lifetime sun exposure. Specifically, we calculated cumulative lifetime UVR for each participant by combining latitude of residence and usual time outdoors obtained from a detailed residency calendar with ambient UVR levels obtained from satellite-derived ground level estimates [19]. The average monthly UVR estimates for each participant were summed from age 6 years to symptom onset/index date.

#### 2.4.2. 25OHD and Genotype

To examine the relationship between the rs7041 or rs4588 genotype and serum 25OHD levels, we used multivariable linear regression with log-transformed 25OHD (normal distribution) as the dependent variable. The models were adjusted for age, sex, BMI, season of 25OHD measurement, genetic ancestry, and case status.

#### 2.4.3. 25OHD, UVR, Vitamin D-Binding Protein Genotype and MS

Multivariable unconditional logistic regression was used to simultaneously examine the independent effects of 25OHD, cumulative lifetime UVR (KJ/m^2^) and the dominant genotypes of rs7041 and rs4588 on MS/CIS by race/ethnicity. 25OHD was log-transformed and both cases’ and controls’ values were deseasonalized by using residuals derived from multivariable linear regression adjusted for season (April–September or October–March) and BMI at 25OHD measurement because BMI had a strong association with 25OHD levels but not MS/CIS risk. The models were adjusted for age, sex, genetic ancestry, smoking and HLA-DRB1*15:01 carrier status.

To determine whether the associations varied by rs7041 or rs4588 genotype, we tested the multiplicative interaction of rs7041 with 25OHD or UVR and rs4588 with 25OHD or UVR separately using the same model as their main effects. These initial models included all participants (blacks, Hispanics and whites) and were adjusted for genetic ancestry, cumulative lifetime UVR and the other pre-specified factors. Because a significant multiplicative interaction (pre-set at *p* < 0.05) was detected for rs7041*25OHD (*p* = 0.016), this model was then stratified by race/ethnicity and dominant rs7041 genotype.

Sensitivity analyses excluding vitamin D supplement users were conducted for all analyses. Two-sample *t-*tests were used to compare means, Wilcoxon–Mann–Whitney test for non-normally distributed variables and χ^2^ or Fisher exact test to compare frequencies between two groups. All analyses were conducted using SAS software v9.3 (SAS Institute, Cary, NC, USA).

## 3. Results

### 3.1. Characteristics of Participants

Table 1 shows the distribution of alleles in two common SNPs in the vitamin D-binding protein gene (rs7041 and rs4588), HLA-DRB1*15:01 (rs3135388), 25OHD levels, cumulative UV dose and the proportions of genetic ancestry in the three racial/ethnic groups, along with demographic characteristics, prevalence of MS risk factors and selected factors that influence 25OHD levels. The allele distribution for these SNPs did not differ significantly between cases and controls in blacks, Hispanics or whites. As anticipated, carrying at least one copy of *HLA-DRB1:15:01* (rs3135388-A allele) was more common in cases than controls particularly in whites and Hispanics.

### 3.2. Polymorphisms in the Vitamin D-Binding Protein Gene and 25OHD

Whites and Hispanics were far more likely than blacks to carry at least one copy of the C allele at rs7041 whereas blacks were more likely than whites or Hispanics to carry an A allele at this location. The A allele at rs7041 was associated with lower 25OHD levels in all three groups but this reached statistical significance only in whites (Table 2). Sensitivity analyses excluding vitamin D supplement users showed similar results (see Appendix A).

The G allele was the dominant allele at rs4588 for all three groups, but whites and Hispanics were less likely to carry this allele and more likely to carry at least one copy of the T allele at rs4588 than blacks. The T allele at rs4588 was associated with significantly lower 25OHD levels in all three groups (Table 2).

### 3.3. Polymorphisms in the Vitamin D-Binding Protein Gene, 25OHD, Cumulative Lifetime UVR and MS

**Figure 1:**
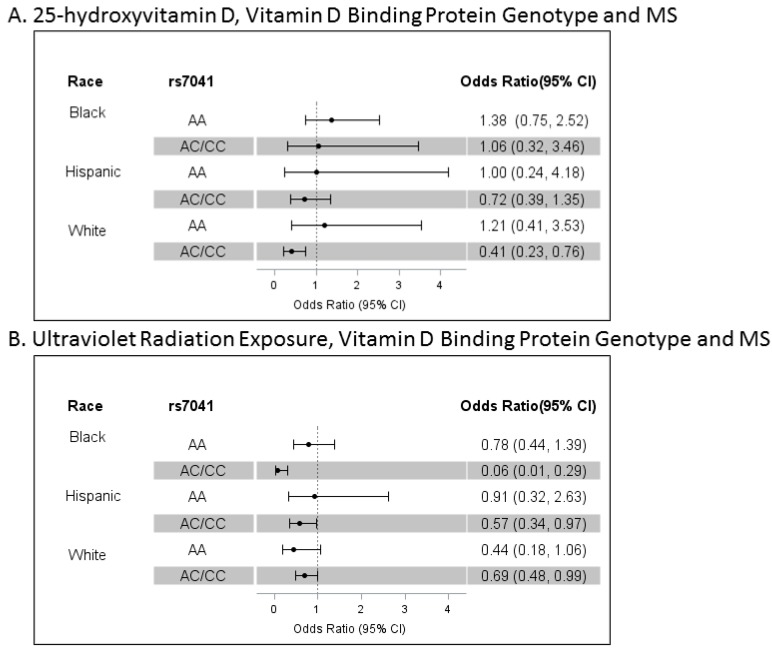
Vitamin D-Binding Protein Genotype, Ultraviolet radiation exposure, 25-Hydroxyvitamin D and MS. Depicted are the odds ratios (ORs) and 95% confidence intervals (CIs) of the independent associations of serum 25-hydroxyvitamin D levels (Panel **A**) or cumulative lifetime ultraviolet radiation exposure (Panel **A**) with risk of multiple sclerosis/clinically isolated syndrome stratified by dominant genotype of SNP rs7041 within each racial/ethnic group. Serum 25-hydroxyvitamin D levels were log transformed and adjusted for BMI and season. Odds ratios are adjusted by age, sex, smoking, genetic ancestry, HLA-DRB1*15:01 (rs3135388) 25-hydroxyvitamin D (Panel **A**) or lifetime ultraviolet radiation exposure (Panel **B**). In blacks, AA is the most common genotype (*n* = 173, 70.0%; AC/CC *n* = 74, 30.0%). Among Hispanics, the distribution is AA *n* = 87, 22.9% and AC/CC *n* = 293, 77.1%; in whites, AA *n* = 108, 21.0% and AC/CC *n* = 406, 79.0%.

An interaction, indicating that higher serum 25OHD levels and carrying at least one copy of the C allele at rs7041 (*p* = 0.016) but not rs4588 was associated with lower MS risk, was detected in models of all participants (blacks, Hispanics and whites pooled) independent of the effects of lifetime UVR exposure, genetic ancestry, rs3135388, sex, age and smoking. In models stratified by race/ethnicity, higher serum 25OHD levels were strongly associated with a lower risk of MS/CIS in whites who carried at least one copy of the C allele at rs7041 (*n* = 406, 79.0%) but not in those homozygous for the A allele. However, no association was found between 25OHD and MS in Hispanics or blacks regardless of rs7041 genotype (Figure 1A).

We did not detect a significant interaction of cumulative UVR and the rs7041 or rs4588 genotype in models that included all participants. However, the protective effect of cumulative UVR did appear to be more pronounced in those carrying at least one copy of the C allele at rs7041. Higher lifetime UVR exposure was associated with a significantly lower risk of MS (independent of 25OHD levels) in blacks and Hispanics who carried at least one copy of the C allele at rs7041 (30.0% and 77.2% respectively) but not in those homozygous for the A allele (Figure 1B). In whites, the magnitude of association between UVR exposure and MS risk was similar in both genotypes but did not reach statistical significance in the smaller group (21%) homozygous for the A allele at rs7041. These findings in whites likely explain the failure to detect a significant interaction between UVR exposure and the rs7041 genotype in models of all participants.

## 4. Discussion

That common polymorphisms in the vitamin D-binding protein gene could modify the relationship between vitamin D and MS risk in blacks and whites is biologically plausible. Vitamin D-binding protein is the main transporter of 25OHD and its metabolites. SNPs in the gene encoding DBP at rs7041 and to a lesser extent rs4588 influence bioavailable fractions because they result in DBP isoforms that have different avidity for 25OHD and its metabolites [9]. The dominant allele at rs7041 in whites is the minor allele in blacks. The resulting DBP isoform most commonly found in blacks has the highest avidity and is the most efficient transporter of 25OHD and its metabolites to target tissues [9]. It is also associated with higher ‘free’ or bioavailable 25OHD levels which may explain why, despite quite low total 25OHD levels, most blacks are not physiologically vitamin D deficient [7]. Following this line of reasoning, we hypothesized that these known racial differences in the prevalence of common genetic polymorphisms in the vitamin D-binding protein gene could explain why we and others [5] have been unable to detect an association between 25OHD and MS risk in blacks.

Yet, the analyses presented herein demonstrate that racial/ethnic variations in bioavailable vitamin D resulting from differences in dominant polymorphisms in the vitamin D-binding protein gene do not explain the lack of association between 25OHD and MS in blacks and Hispanics. Taken together with our previously reported findings that higher levels of UVR exposure from sunshine are independently and consistently associated with lower MS risk across all 3 racial/ethnic groups [7], these findings further challenge the biological plausibility of vitamin D deficiency as causal for MS.

While it may be tempting to cling to the idea that somehow vitamin D is causally linked to MS risk in whites but just not in blacks and Hispanics, it is difficult to conceive how a factor could be causal in only one racial/ethnic group. One could contend that the consistently protective effect of UVR across all three groups is still ultimately mediated by vitamin D, but that serum 25OHD is the wrong measure of vitamin D status in blacks and Hispanics. However, there are several problems with this line of reasoning: (1) the common polymorphisms we measured are in coding regions and directly influence the bioavailable fraction of 25OHD and its metabolites; (2) the prevalence of these common polymorphisms in whites and Hispanics are almost identical yet we cannot establish a relationship between MS risk and 25OHD in Hispanics. Furthermore, other studies of Hispanics with MS have reported that 25OHD levels do not differ between prevalent cases or controls [20], or decline with increasing disability as has been shown in whites [21]; (3) There is increasing evidence that UVR causes a plethora of immunological changes including effects that could influence autoimmune diseases independent of vitamin D. These include generation of T regulatory cells, B suppressor (regulatory) cells and production of immunosuppressive lipid mediators and alarmins [22] as well as suppression of the animal model of MS (experimental autoimmune encephalomyelitis) [23,24,25]. Lastly (4), the amount of UVR exposure, while consistently associated with MS risk, is strongly related to latitude of residence. It is important to remember that the vitamin D-MS hypothesis originated from the observation that the prevalence of MS is higher at latitudes further away from the equator where the intensity of UVR exposure is lower. However, UVR may not be the only reason for the observed latitude gradient as there are other potential MS risk factors that traditionally varied with latitude, including endemic infectious agents, food sources and genetic make-up of native inhabitants.

Our data are consistent with previous studies that examined the relationship between vitamin D-binding protein genotype and serum 25OHD levels in blacks, Hispanics and whites [6,7,22,23,24]. We found similar distributions of rs7041 and rs4588 polymorphisms and similar associations of these genes with lower 25OHD levels across all three groups [7,22,23,24]. Our findings are also consistent with the results of randomized controlled trials that have failed to demonstrate convincing health benefits of vitamin D supplementation [26,27,28] in other diseases.

Intriguingly, we also found that the strong association of higher sun exposure and lower MS risk in blacks, Hispanics and whites is most pronounced in those individuals who carry at least one copy of the C allele at rs7041. This difference is most noticeable in blacks even though only 30.4% carry at least one C allele at this location. We think this stronger effect in blacks may be because they are less likely to use sunscreen than whites; thus blacks effectively get more sun exposure for the same amount of time outdoors at a particular ambient UVR than whites. We cannot exclude the possibility that this is a chance finding, as we did not detect a significant multiplicative interaction between UVR and rs7041 in the pooled cohort where only genetic ancestry and not self-reported race/ethnicity was considered. Rather, this association was uncovered when we examined the results of the stratified models that were run after detecting an interaction between 25OHD and rs7041 in the pooled cohort.

Alternatively, we speculate that the different DBP isoforms may have novel interactions with the immunomodulatory effects of UVR independent of vitamin D. Aside from transporting vitamin D, DBP also directly influences macrophage and neutrophil function and plays a role in tissue injury and repair [10]. Only 5% of the binding sites on DBP are occupied by vitamin D, implying that this protein’s major role may be through other actions [10]. UVR exposure results in immunomodulation through multiple mechanisms, many of which are independent of vitamin D. Such a non-classical interaction between DBP and UVR would be a more satisfying unifying explanation of our findings. Yet the evidence supporting such an interaction is lacking as no studies directly address the role of DBP in photoimmunology and very few studies have examined whether specific DBP genotypes are associated with differences in other DBP functions such as macrophage activation [10].

If this finding can be replicated, it would have wide-reaching implications for the role of vitamin D in MS and other disease states as it implies that variation in the DBP genotype may affect MS risk through a vitamin D-independent pathway. Such a finding would invalidate the current instruments for Mendelian randomization studies of vitamin D because it violates the assumption that the genotypes affect the risk of disease only through influencing vitamin D status. This has important implications for the MS-vitamin D hypothesis, as Mendelian randomization studies are the strongest evidence to date to support a causal relationship between 25OHD and MS risk in whites. The main limitation of this study is that we were unable to identify an existing cohort of blacks or Hispanics with incident MS and sun exposure, 25OHD measurements and genotype in which these findings could be replicated. We also cannot exclude the possibility that we are underpowered to detect a protective association with 25OHD and MS in the small subgroups of white or Hispanic AA carriers and black carriers of AC/CC. Other limitations of this study include the case-control design necessitating that most 25OHD measures were obtained after symptom onset (although very close to the time of diagnosis). MS often results in sun avoidant behavior due to heat sensitivity which can cause an *exaggerated* association between low 25OHD and MS particularly in prevalent cases. This most likely explains the observed lower 25OHD levels in a previous study of prevalent black MS cases compared to controls [29] and may explain the strong association we found in whites, but does not explain the *lack* of association we found in Hispanics and blacks. Likewise, multiple statistical tests would be expected to lead to false positive results rather than consistently negative findings. Data on sunscreen use over the life course were limited. Sunscreen is more likely to have been used by whites than other racial groups (since they are more sun sensitive), and regular and correct use of a high SPF sunscreen would reduce the effective UVR exposure of the skin. Thus, our effect estimates presented herein likely underestimate the protective effect of the received UVR dose in whites.

This study highlights how multi-ethnic studies can lead to novel insights into disease. Important racial variations in the gene encoding DBP do not explain the lack of association between 25OHD and MS risk in blacks and Hispanics, yet higher levels of UVR may be particularly important in protecting against the risk of MS in those carrying the DBP dominant in whites. Replication of our key findings should be addressed in future studies.

## Figures and Tables

**Table 1:** Selected characteristics at symptom onset/index date of MS/CIS cases and controls by race/ethnicity.

	Black (*n* = 247)			Hispanic (*n* = 380)			White (*n* = 514)		
	Case (*n* = 116)	Control (*n* = 131)	*p* *	Case (*n* = 183)	Control (*n* = 197)	*p* *	Case (*n* = 247)	Control (*n* = 267)	*p* *
Age, mean (SD), y	38.4 (12.8)	38.5 (13.0)	0.93	32.5 (10.7)	32.6 (11.1)	0.95	39.7 (12.0)	39.9 (12.2)	0.87
Female, *n* (%)	92 (79.3)	103 (78.6)	0.90	132 (72.1)	145 (73.6)	0.75	164 (66.4)	174 (65.2)	0.77
Smoking, *n* (%)	27 (23.3)	33 (25.2)	0.73	44 (24.0)	38 (19.3)	0.26	115 (46.6)	90 (33.7)	0.003
BMI, mean (SD)	30.5 (7.4)	31.6 (8.1)	0.26	29.5 (6.8)	30.1 (7.0)	0.42	28.5 (6.7)	28.5 (6.9)	0.95
VitD supplement users *, *n* (%)	28 (24.1)	2 (1.5)	<0.001	30 (16.4)	3 (1.5)	<0.001	62 (25.1)	19 (7.1)	<0.001
Season **, *n* (%)	56 (48.3)	65 (49.6)	0.83	85 (46.5)	104 (52.8)	0.22	116 (47.0)	142 (53.2)	0.16
cumulative UV dose ***, 1000 KJ/m^2^, mean (SD)	1.43 (0.77)	1.63 (0.79)	0.05	1.14 (0.61)	1.22 (0.68)	0.22	1.53 (0.79)	1.64 (0.83)	0.15
serum 25OHD, nmol/L, median (IQR)	43.7 (32.4,74.9)	47.4 (30.0,64.9)	0.53	54.9 (42.4,69.9)	57.4 (44.9,69.9)	0.35	67.4 (49.9,87.4)	72.4 (59.9,92.4)	0.003
Vitamin D-Binding Protein									
rs7041, *n* (%)			0.53			0.64			0.37
AA	79 (68.1)	94 (71.7)		40 ( 21.9)	47 (23.8)		56 (22.7)	52 (19.4)	
AC	32 (27.6)	31 (23.7)		93 (50.8)	100 (50.8)		116 (47.0)	123 (46.1)	
CC	5 (4.3)	6 (4.6)		50 (27.3)	50 (25.4)		75 (30.3)	92 (34.5)	
rs4588, *n* (%)			0.13			0.52			0.44
GG	96 (82.8)	98 (74.8)		110 (60.1)	112 (56.8)		122 (49.4)	141 (52.8)	
GT	18 (15.5)	29 (22.1)		66 (36.1)	75 (38.1)		105 (42.5)	99 (37.1)	
TT	2 (1.7)	4 (3.1)		7 (3.8)	10 (5.1)		20 (8.1)	27 (10.1)	
HLA-DRB1*1501, *n* (%)			0.20			0.0002			<0.0001
GG	95 (81.9)	115 (87.8)		134 (73.2)	174 (88.3)		136 (55.1)	214 (80.1)	
AG	21 (18.1)	15 (11.5)		47 (25.7)	19 (9.7)		92 (37.2)	52 (19.5)	
AA	0 (0.0)	1 (0.8)		2 (1.1)	4 (2.0)		19 (7.7)	1 (0.4)	
Ancestry % median (IQR)									
European	21.2 (14.6, 28.0)	22.8 (16.3, 31.9)		58.6 (50.1, 66.0)	56.6 (48.5, 64.3)		99.0 (97.6, 99.7)	99.1 (97.6, 99.8)	
African	77.6 (69.8, 84.4)	75.4 (66.2, 83.0)		5.1 (3.8, 6.9)	4.9 (3.8, 6.4)		0.1 (0.0, 0.9)	0.0 (0.0, 0.8)	
Amerindian	0.3 (0.1, 1.4)	0.7 (0.2, 1.9)		35.7 (27.0,42.8)	38.0 (31.0, 46.1)		0.5 (0.2, 1.1)	0.4 (0.2, 1.2)	

MS = multiple sclerosis, CIS = clinically isolated syndrome, SD = standard deviation, IQR = interquartile range, BMI = body mass index, VitD = vitamin D, UV = ultraviolet radiation; y = years. * At the time of 25OHD measurement; ** season (April–September) at the time of 25OHD measurement; *** from age 6 years to index date.

**Table 2:** Association of vitamin D-binding protein genetic polymorphisms and serum 25OHD levels by race/ethnicity.

Race/Ethnicity	SNP	*n*	Reference Allele	Variant Allele	Variant Allele Frequency	β Estimate *	Change in Total 25OHD Level Per Variant Allele Copy (95% CI), nmol/L	*p*
Black	rs7041	247	C	A	0.828	−0.1136	−5.5 (−11.9 to 0.2)	0.0573
Hispanic	rs7041	380	C	A	0.483	−0.0492	−2.7 (−5.9 to 0.3)	0.0757
White	rs7041	514	C	A	0.443	−0.0813	−5.9 (−9.3 to −2.7)	0.0002
Black	rs4588	247	G	T	0.119	−0.2148	−8.9 (−13.5 to −3.7)	0.0014
Hispanic	rs4588	380	G	T	0.230	−0.0836	−4.4 (−7.6 to −0.9)	0.0135
White	rs4588	514	G	T	0.290	−0.1160	−7.7 (−10.5 to −4.7)	<0.0001

25OHD = 25-hyrdoxyvitamin D, CI = confidence intervals; * adjusted for age, sex, BMI, season of 25OHD measurement, smoking, genetic ancestry and case status.

## Appendix A

### Appendix A.1. Supplementary Methods

#### Appendix A.1.1. Study Population, Case and Control Identification and Recruitment

KPSC provides comprehensive health care to ~20% of the population in the geographic area it serves [11]. To identify potentially eligible cases, we searched the complete electronic health record (EHR) electronically monthly for first mention of ICD-9 diagnostic codes for MS or CIS 1 January 2011–31 December 2014. Diagnoses were confirmed by an MS specialist (ALG) after complete EHR review according to revised McDonald criteria for MS [14] and consensus definitions for idiopathic transverse myelitis (TM) [15,16]. All diagnoses of optic neuritis (ON) were confirmed by ophthalmologists. Symptom onset date and date of diagnosis were validated by structured interview. Patients who met diagnostic criteria for neuromyelitis optica spectrum disorder [30] were excluded. Due to resource limitations and because the primary goal was to study non-white populations, recruitment of white cases was stopped after the target of *n* = 250 had been reached. Study subjects of Asian/Pacific Islander (56 subjects) and Multiple (2 subjects) races were excluded from the analyses due to small sample size. We plan on examining Asian/Pacific Islanders separately once we have recruited sufficient number of participants as we expect the effects of vitamin D and sun exposure on the risk of MS to differ from blacks and whites.

There are slightly more controls in the MS Sunshine Study than cases primarily because sometimes the race/ethnicity indicated in the EHR did not match the participant’s self-reported race/ethnicity. This mis-match between the EHR and self-reported race/ethnicity occurred primarily with white, Hispanics and white, non-Hispanics. To identify eligible controls matched to cases on race/ethnicity, race/ethnicity was initially pulled electronically from the EHR. If, during the structured in-person interview, the control reported a different race/ethnicity than their respective case, another control was recruited for the case until a true racial/ethnic match was completed. Asians (total cases and controls *n* = 57) and people of multiple races (*n* = 2) were recruited but not included in the analyses due to small sample.

#### Appendix A.1.2. Genotyping

The genotyping data was available on 1159 (97.6%) of the 1187 black, Hispanic or white subjects who had completed the study protocol by 6/2/2015. Eighteen subjects were excluded due to missing phenotype data as follows: 5 due to missing serum 25(OH) D values; 11 for missing cumulative UVR data; and 2 for missing smoking status. The final analysis cohort included 1141 (96.1%) subjects: 247 Blacks (116 cases/131 controls), 380 Hispanics (183 cases/197 controls) and 514 whites (247 cases/267 controls).

These subjects were genotyped successfully for 697,895 SNPs using Illumina’s HumanOmniExpressExome v1.2. DNA Analysis BeadChips were produced by the Vincent J. Coates Genomics Sequencing Laboratory (GSL) at the University of California, Berkeley. DNA samples were quantitated using the Nanodrop ND-1000 and subsequently normalized and plated for processing. The samples were processed using the Illumina Infinium HD Assay Super protocol. DNA samples were denatured, neutralized, and prepared for amplification. The amplified product was then fragmented, precipitated, and collected by centrifugation. The precipitated DNA was resuspended in hybridization buffer and subsequently hybridized to a beadchip. The beadchip was then prepared for extension and staining. Once the assay was completed, the chips were dried and placed on the scanner for data collection. Data was then QC’d using Genome Studio and Plink.

From Within GenomeStudio, various QC measures were checked including call rates, sex discrepancies, reproducibility and heritability of replicates and CEPH control trios, as well as performance of internal Illumina controls. Any samples with the above discrepancies were noted down. Samples with call rates of less than 90% or less than 99% were also noted. In addition, the following data from PLINK1.07 [18] were filtered: *N*/percent of SNPs with call-rate <90%, *N*/percent of SNPs with Hardy Weinberg (all samples) *p*-value < 1 × 10^−6^, *N*/percent of SNPs with MAF < 0.01.

A summary of Control target intensities was then created to evaluate the performance of Illumina’s internal controls. Illumina includes several control targets for the purpose of QC at various stages. The Staining, Extension, Target Removal, and Hybridization controls are all sample-independent measures that show the performance of the assay. The Stringency, Non-Specific Binding and Non-Polymorphic controls are all sample-dependent controls.

We performed sex check and tested the deviation from Hardy–Weinberg equilibrium within racial/ethnic group on SNPs using PLINK 1.07.

### Appendix A.2. Ancestry Structure

To investigate genetic ancestry, the software STRUCTURE Version 2.3.1 [17] was used to infer the presence of distinct populations. A genome-wide set of 67,547 linkage disequilibrium pruned loci were selected using PLINK. We compared the structure outputs from three (Europeans, Africans, and Amerindians), five (Europeans, Africans, Amerindians, East Asians, and Central/South Asians), and seven (Europeans, Africans, Amerindians, East Asians, Central/South Asians, Western Asians and Oceanians) reference populations, and concluded that using five or seven reference populations did not improve upon the three population model for estimating population admixture in our cohort. With three populations assumed, the probability of population ancestry was estimated by specifying a 10,000 iteration burn-in period and a 10,000 iteration follow-up of the Markov Chain Monte Carlo model utilized by STRUCTURE.

### Appendix A.3. Deseasonalized 25OHD Values for Other Analyses

For analyses that include unmatched cases and controls, 25OHD levels were deseasonalized for both cases and controls and did not result in significantly different findings. Seasons were combined into two seasons, winter (October–March) and summer (April–September) based on the inflection points of the best-fit polynomial regression (quadratic function) of the monthly variation of 25OHD values among controls. This is consistent with Southern California’s annual fluctuation in temperatures and daylight hours. We did not detect significant variation between fall and winter or spring and summer.

### Appendix A.4. Cumulative Lifetime UVR

Cumulative lifetime UVR for each participant was calculated using previously validated methods [1]. Average monthly estimates derived by linking latitude and longitude of residence with satellite-derived ground level estimates of ambient UVR multiplied by proportion of time in the sun during summer were summed from age 6 years to symptom onset/index date. Specifically, we used the proportion of self-reported hours outside in the sun for weekends (26 days) and holidays (14 days) in the season (summer: (sun time in hour/12) * 40 to calculate the personal cumulative leisure-time UVR dose in summer for each participant. The leisure-time UVR dose was calculated as ambient UV x proportion of days in the sun, then summed over the relevant period, from 6 years of age until symptom onset/index date.

### Appendix A.5. Supplementary Results

**Table A1:** Sensitivity analysis: Association of Vitamin D-binding protein Genetic Polymorphisms and serum 25OHD levels by race/ethnicity excluding subjects using vitamin-D supplements at the time of 25OHD measurement.

Race/Ethnicity	SNP	*n*	Reference Allele	Variant Allele	Variant Allele Frequency	Β Estimate *	Change in Total 25OHD Level Per Variant Allele Copy (95% CI), nmol/L	*p*
Black	rs7041	217	C	A	0.825	−0.1322	−6.3 (−13.1 to −0.4)	0.0358
Hispanic	rs7041	347	C	A	0.488	−0.0490	−2.8 (−5.9 to 0.3)	0.0733
White	rs7041	433	C	A	0.441	−0.0675	−4.9 (−8.4 to −1.5)	0.0044
Black	rs4588	217	G	T	0.124	−0.2290	−9.2 (−13.7 to −4.1)	0.0009
Hispanic	rs4588	347	G	T	0.232	−0.0933	−4.9 (−8.1 to −1.5)	0.0052
White	rs4588	433	G	T	0.281	−0.1191	−7.9 (−10.9 to −4.6)	<0.0001

25OHD = 25-hyrdoxyvitamin D, CI = confidence intervals; * adjusted for age, sex, BMI, season of 25OHD measurement, smoking, genetic ancestry and case status.
